# Impairment of circulating endothelial progenitor cells (EPCs) in patients with glucocorticoid-induced avascular necrosis of the femoral head and changes of EPCs after glucocorticoid treatment in vitro

**DOI:** 10.1186/s13018-019-1279-6

**Published:** 2019-07-19

**Authors:** Peng Ding, Wen Zhang, Qiyuan Tan, Chen Yao, Sen Lin

**Affiliations:** 1 Department of Orthopaedics, Shanghai Jiaotong University Affiliated Shanghai Sixth People’s Hospital, Shanghai, 200233 People’s Republic of China; 2 Department of Endocrinology, Shanghai Jiaotong University Affiliated Shanghai Sixth People’s Hospital, Shanghai, 200233 People’s Republic of China

**Keywords:** Endothelial progenitor cells, Glucocorticoid, Glucocorticoid-induced ANFH

## Abstract

**Background:**

Avascular necrosis of the femoral head (ANFH) is a severe complication after high-dose glucocorticoid (GC) administration. The pathogenesis of GC-induced ANFH remains unclear. Though the important role of endothelial progenitor cells (EPCs) in the progression of GC-induced ANFH has been noticed, the effects of GCs on EPCs and the underlying mechanism still need further study.

**Methods:**

Circulating EPCs were obtained from the peripheral blood of ANFH patients and healthy controls by Ficoll-density gradient centrifugation. CD133^+^CD34^+^ cells with DiI-Ac-LDL uptake and FITC-UEA-1 binding were considered as EPCs. Number and functions of EPCs were analyzed by flow cytometry, chemotaxis assay, and tube formation assay. EPCs from healthy controls were also treated by different concentrations of methylprednisolone and prednisolone in vitro, and cell growth and angiogenic function were evaluated. Expression of CXCR7 and its downstream Akt/GSK-3***β***/Fyn pathway were also analyzed by western blots after cells treated by methylprednisolone in vitro.

**Results:**

The number and functions of EPCs in patients with GC-induced ANFH were significantly decreased. In vitro study showed for the first time that except extremely high concentrations, low to medium concentrations of GCs did not have significant effects on EPCs’ growth. Methylprednisolone and prednisolone both inhibited angiogenesis of EPCs even at low concentrations. Mechanism studies found CXCR7 was downregulated in EPCs after methylprednisolone treatment in vitro. Expression and phosphorylation of Akt and GSK-3***β*** were also decreased with an upregulation of Fyn expression after steroid treatment.

**Conclusions:**

Our study showed that GC-induced ANFH patients have reduced the number and impaired functions of circulating EPCs. GCs did not show a significant effect on the growth of EPCs in vitro except extremely high concentrations of GCs. However, GCs significantly impaired EPC angiogenic function in vitro, even at low concentrations. Our study also suggested that downregulation of CXCR7 and its downstream Akt/GSK-3***β***/Fyn pathway in EPCs might be a novel mechanism of how GCs suppress EPCs’ angiogenesis.

## Background

Avascular necrosis of the femoral head (ANFH) is a progressive disease characterized by necrosis and collapse of the bone. ANFH often ends in arthroplasty and has become a severe health problem worldwide. High-dose of GC administration is a common risk factor associated with ANFH due to a widespread use of GCs as adjuvant therapies in the treatment of many inflammatory and autoimmune diseases. Though the specific etiology of ANFH remains unclear, the pathophysiology of impaired circulation in the femoral head has been considered as one major cause of ANFH in patients [1]. In GC-induced ANFH, insufficient neovascularization and elevated vascular permeability in the osteonecrotic lesions have been demonstrated [2]. Based on this observation, many hypotheses have been proposed to address the pathogenesis of ANFH, such as apoptosis, oxidative stress, disorders of the vascular endothelium, blood coagulation disorders, fat embolism, and multiple-hit theory. However, none of these reached consensus, so far.

In 1997, the endothelial progenitor cell (EPC) was first reported by Asahara and colleagues [3]. They described EPCs as a group of circulating cells which promoted angiogenesis and maintained vascular homeostasis. The role of EPCs in certain diseases has also been studied. The level of EPCs predicts the occurrence of cardiovascular events and may help to identify patients with a high risk of cardiovascular diseases [4]. In ANFH, Sun et al. [5] found transplantation of EPCs promoted vascularization and bone regeneration in the early stage of ANFH in a rabbit model. Though the anti-inflammatory effect of GCs helps to stabilize the endothelium, the effects of GCs on endothelial progenitor cells are still controversial. Grisar et al. [6] found loss of EPC function and number in SLE patients and dexamethasone treatment significantly increased patients’ EPC population and CFU formation. Feng et al. [7] found EPCs’ number was reduced in ANFH patients with different risk factors including the use of steroids. Paradoxically, Chen et al. [8] reported an unchanged number of circulating EPCs in GC-induced ANFH patients. Moreover, as published literatures discussing GCs’ effect on EPCs were mostly based on in vivo studies, the direct effect of GCs on circulating EPCs in vitro has never been extensively studied. The change of EPCs in ANFH patients and the mechanisms of how GCs affect EPCs’ population and function still need further investigation.

In the current study, we investigated the population and function of circulating EPCs in ANFH patients by using a combination of EPC markers different from published literatures. In addition, we also studied and compared the effects of methylprednisolone and prednisolone on EPCs in vitro. We demonstrated for the first time that GCs showed significant inhibitory effect on EPC function of angiogenesis in vitro, while the growth of EPC was only inhibited under extremely high GC concentrations. We also demonstrated that steroids significantly downregulated CXC chemokine receptor 7 (CXCR7), which may be an important mechanism of suppressed EPCs’ angiogenesis after GC treatment in vitro.

## Methods

### Participant selection

Between March 2016 and September 2017, 10 adult male patients with glucocorticoid-induced ANFH (average age 37.97 years, ranging from 20 to 40 years) were recruited in the authors’ department. Ten healthy volunteers with similar age and body weight (average age 38.73 years, ranging from 20 to 40 years) were also recruited as a control group. Clinical backgrounds of all participants were summarized in Table 1. GC-induced ANFH was diagnosed by a long-term steroid use history, complaint of hip pain, and positive X-ray and magnetic resonance imaging [1, 9]. Exclusion criteria included age under 18 years old, cancer, pregnancy, diabetes mellitus, current and previous bone infections, immunosuppressive drug therapy, history of inflammatory arthritis, cardiovascular diseases, impaired renal function, and mental health problems [10, 11].

**Table 1:** Clinical characteristics of study subjects

Characteristics	ONFH (*n* = 30)	Control (*n* = 30)
Age (years)	37.97 ± 8.99	38.73 ± 8.18
Body mass index (kg/m^2^)	24.39 ± 4.00	23.67 ± 4.46
Systolic BP (mmHg)	120.37 ± 13.12	109.57 ± 8.96
Diastolic BP (mmHg)	79.40 ± 10.78	71.73 ± 7.07
Blood glucose (mmol/L)	5.00 ± 0.48	4.99 ± 0.43

Data are mean ± standard derivation. *BP* blood pressure

### Isolation and culture of circulating EPCs

Peripheral blood sample (20 ml) was collected and centrifuged using the Ficoll-density gradient method. Buffy coat mononuclear cells (MNCs) were collected and washed with HANKs’ balanced salt solution twice. MNCs were then resuspended with EGM-2 medium (Lonza, San Diego, CA) and plated on human fibronectin-coated plates (BD Biosciences, Bedford, MA) at a density of 4 × 10^6^ cells. Culture medium was changed every 3 days, and passage was performed when cell colonies were formed [12, 13].

### Characterization of EPCs

For flow cytometry, EPCs on day 7 were detached and resuspended in PBS. After Fc-blocking, 1.0 × 10^6^ cells were incubated with conjugated anti-CD133-PE (BD Biosciences, San Jose, CA) and CD34-FITC (BD Biosciences, San Jose, CA) for 20 min at 4 °C. Cells were analyzed using Beckman Coulter FC500 flow cytometer. To observe DiI-ac-LDL uptake and FITC-UEA-1 binding, EPCs were incubated with 2.4 μg/mL Dil-Ac-LDL (Invitrogen-Molecular Probes, Eugene, OR) in EGM-2 medium at 37 °C for 4 h. Then, cells were fixed in 4% paraformaldehyde and further incubated with 20 μg/mL FITC-UEA-1 (Introvogen-Molecular Probes, Eugene, OR) for 1 h. For immunofluorescence staining, EPCs were fixed in 4% paraformaldehyde for 10 min, blocked in 5% normal goat serum, and then incubated with anti-CD133-PE (BD Biosciences, San Jose, CA) and anti-CD34-FITC (BD Biosciences, San Jose, CA) antibodies at 4 °C overnight. Fluorescence was observed under a confocal laser scanning microscope.

### Migration assay

Migration assay was performed on the Zigmond chambers (Neuro Probe, Gaithersburg, MD). Twenty microliters of complete EGM-2 medium with EPCs from patients or healthy controls were seeded in the left Zigmond chamber and 20 μl complete EGM-2 medium containing only SDF-1α (200 ng/ml) was added to the right chamber. Cell migration was observed during the next 24-h culture and photographs were recorded every 5 min by a digital camera mounted on a light microscope. Migration distances of the cells were observed and quantified by using the Leica Q-Win software.

### Cell cytotoxicity assay

The Cell Counting Kit-8 (CCK-8) assay was used to determine the effect of GCs on EPCs. EPCs were seeded at a density of 1 × 10^3^ cells/well in a 96-well plate in triplicates and a series of concentrations (0.4, 4, 40, 400, and 4000 μg/ml) of GCs (prednisolone or methylprednisolone) were applied to the culture medium. After a 72-h culture, CCK-8 assay was performed according to the manufacturer’s instructions. Absorbance was observed at a wavelength of 450 nm by a microplate reader (Multiskan MK3; ThermoFisher Scientific, Waltham, MA).

### In vitro tube formation assay

Matrix Matrigel (BD Biosciences, San Jose, CA) was prepared according to the manufacturer’s instructions. After a 7-day culture, EPCs were collected and plated at a density of 5 × 10^4^/well on top of Matrigel in a 96-well plate in triplicates. Cells were cultured with a series of concentrations (0, 0.4, 4, 40, and 400 μg/ml) of GCs (prednisolone or methylprednisolone) in the medium for 3 days. The diameters of closed network units formed by cells in six continuous visual fields were counted and analyzed by Leica Q-Win software.

### Western blot assay

Normal EPCs were treated with 40 μg/ml methylprednisolone or the same volume of PBS for 24 h before harvest using a regular lysis buffer containing protease inhibitor. The total protein concentration was measured by Bradford assay (BioRad, CA, USA) following the instructions of the manufacturer. Thirty micrograms of protein of each sample was separated on a 10% sodium dodecyl sulfate-polyacrylamide gel electrophoresis (SDS-PAGE) and transferred onto a PVDF membrane (EMD Millipore, Darmstadt, Germany). An immunoblotting analysis was performed. Briefly, the membranes were blocked with 5% Bovine Serum Albumin (Sigma-Aldrich, Missouri, USA) in Tris-buffered saline with 0.5% Tween 20 (TBST) for an hour at room temperature, then incubated with the appropriate primary antibody overnight. Primary antibodies were diluted as instructed by the manufacturers. Primary antibodies include anti-GAPDH (PTG, Rosemont, USA), anti-CXCR7 (Abcam, Cambridge, UK), anti-GSK3**β** (Abcam, Cambridge, UK), anti-phospho-GSK3**β** (Abcam, Cambridge, UK), anti-AKT (Cell Signaling Technology, Massachusetts, USA), anti-phospho-AKT (Abcam, Cambridge, UK), and anti-FYN (Abcam, Cambridge, UK). The membranes were washed with TBST buffer and then incubated with HRP-conjugated anti-rabbit or anti-mouse secondary antibody for an hour at room temperature. Then, the membranes were washed and exposed to the enhanced chemiluminescence.

### Statistics

All data were expressed as mean values ± standard derivation with a 95% confidence interval. Statistical analysis was performed by paired Student’s *t* tests (two-tailed) or variance analysis using a SPSS 13.0 software and *P* < 0.05 was considered significant.

## Results

### Clinical characteristics of participants

Clinical characteristics of ANFH patients and healthy controls were compared and summarized in Table 1. Background factors including age, sex, body mass index (BMI), and blood glucose were comparable between the two groups with no statistical difference. Though the average blood pressure (BP) level of ANFH patients was slightly higher than the healthy controls, the BP levels of both group were within the normal range.

### Characterization of EPCs

**Fig. 1:**
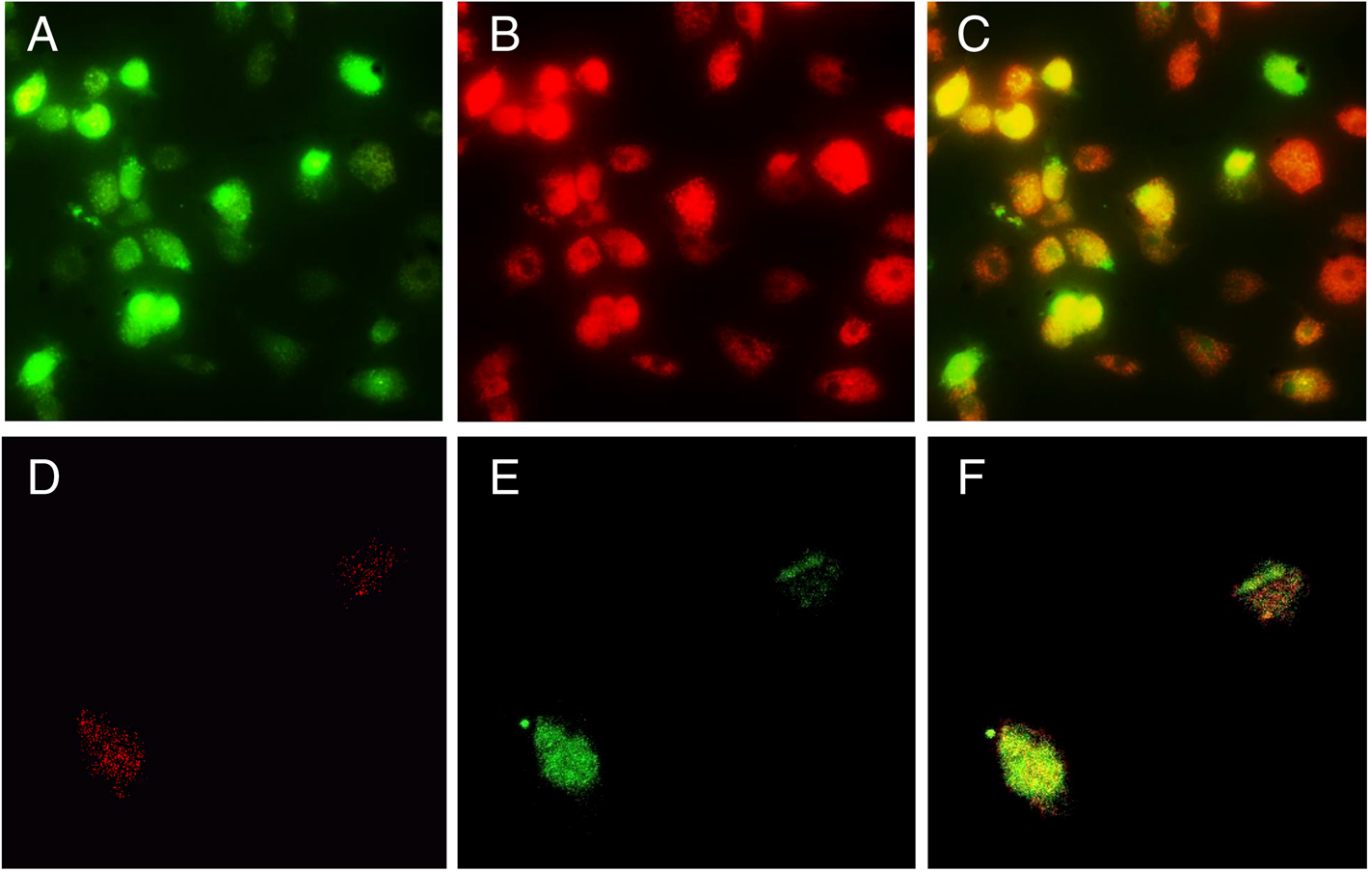
Characterization of human EPCs. **a**–**c** EPCs stained positively for DiI-ac-LDL (**a**) and FITC-UEA-1 (**b**). **c** Merged. **d**–**f** EPCs stained positively for CD133 (**d**) and CD34 (**e**). **f** Merged

**Fig. 2:**
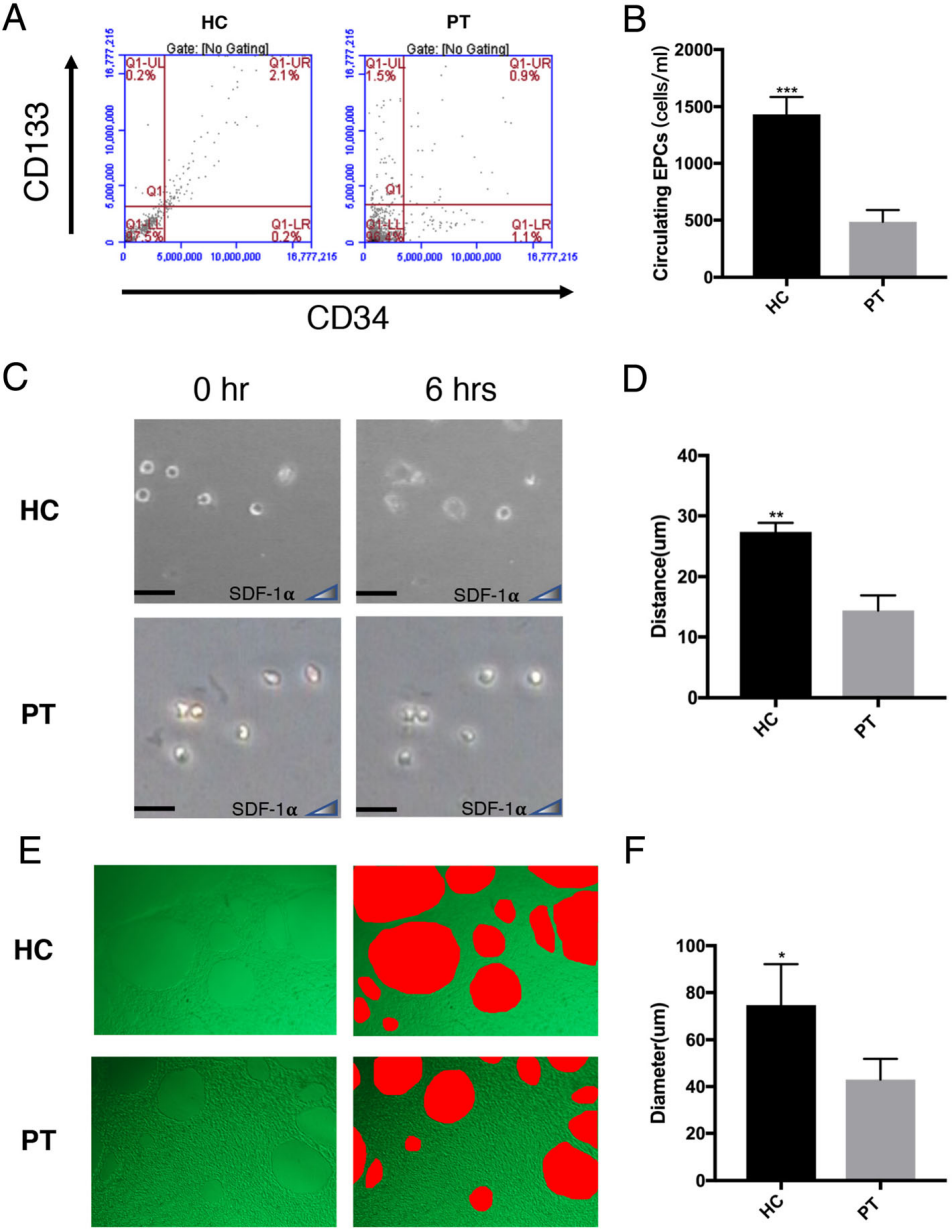
EPCs from glucocorticoid-induced ANFH patients had decreased cell number and functions. **a**, **b** GC-induced ANFH patients have less circulating EPCs in peripheral blood. **a** Representative diagrams of flow cytometry analysis of CD34^+^CD133^+^ cells from healthy controls (left panel) and patients (right panel). **b** Quantification of EPC numbers from the two groups. **c**, **d** Chemotaxis assay showed EPCs from GC-induced ANFH patients had impaired function of migration. **c** Representative images of cells incubated in Zigmond chamber for 0 and 6 h as indicated. Cells’ mobilization towards SDF1 and lamellipodia formation was observed in the control group (HC, upper panels) but not in patient group (PT, lower panels). Bar = 10 μm. **d** Average cell moving distance in the two groups after a 24-h incubation. **e**, **f** Tube formation assay showed EPCs from GC-induced ANFH patients had impaired function of angiogenesis. **e** Representative images of tube formation. Left panels, images with no highlights. Right panels, images with highlights outlining the tubules. **f** The average diameter of tubules formed by cells in each group. HC healthy controls, PT patients; *p < 0.05, **p < 0.01, ***p < 0.001

After a 3-day culture, cells were spindle-shaped under a light microscope. At day 7, EPCs were stained positively for both DiI-Ac-LDL and FITC-UEA-1 binding (Fig. 1a–c). At the same time, EPCs were also stained positively for both CD34 and CD133 under confocal microscopy and flow cytometry (Fig. 1d–f; Fig. 2a). After a 2-week culture, spindle-shaped cells turned into “cobblestone” morphology which is a typical pattern of endothelial cells. The study of cell morphology and markers suggested that purification and in vitro culture of circulating EPCs were successful.

### GC-induced ANFH patients had less circulating EPCs in peripheral blood

Monocytes of ANFH patients and healthy control group were separated from peripheral blood by Ficoll-gradient centrifugation, cultured for 7 days, and stained with CD133 and CD34 antibodies. The CD133^+^CD34^+^ cells were considered as EPCs and the population was analyzed by flow cytometry. Our results showed that the patient group had significantly less EPCs than the control group (Fig. 2a, b). It has been reported that in GC-induced ANFH patients, circulating EPC number may be decreased or remain unchanged when combinations of VEGFR2/CD133 or VEGFR2/CD34 were used to analyze the population [7, 8]. By using a different combination of EPC markers, our study further confirmed that the number of circulating EPCs in GC-induced ANFH patients was lower than healthy controls.

### EPCs from GC-induced ANFH patients had impaired functions of migration and angiogenesis

Besides the number of EPCs, the functions of EPCs may also be compromised after steroid treatment [14, 15]. To compare the migratory function of EPCs, chemotaxis assay was performed using the Zigmond chamber with SDF-1α as chemo-attractant. Instead of counting the number of cells migrated down through the pores of transwells [7, 8], Zigmond chamber allows direct visualization and accurate quantification of cells’ migration distances and traces. EPCs were incubated for a total of 24 h and their migration traces and distances were analyzed. After a 6-h culture, cells moving towards SDF-1α as well as the formation of lamellipodia were observed in the control group. In the patient group, there were only minor movements of cells with no obvious morphological change (Fig. 2c). At the end of the experiment, the average migrated distance of patients’ EPCs was significantly shorter than the healthy control group (Fig. 2d).

Neovascularization assay performed on Matrigels also showed that after 72-h culture, the average diameter of the tubules formed by patients’ EPCs was significantly smaller than the control group (Fig. 2e, f). These results suggested that the angiogenic and migratory functions of EPCs were both significantly impaired in patients with steroid-induced avascular necrosis of femoral head.

### Effects of GCs on EPCs in vitro

**Fig. 3:**
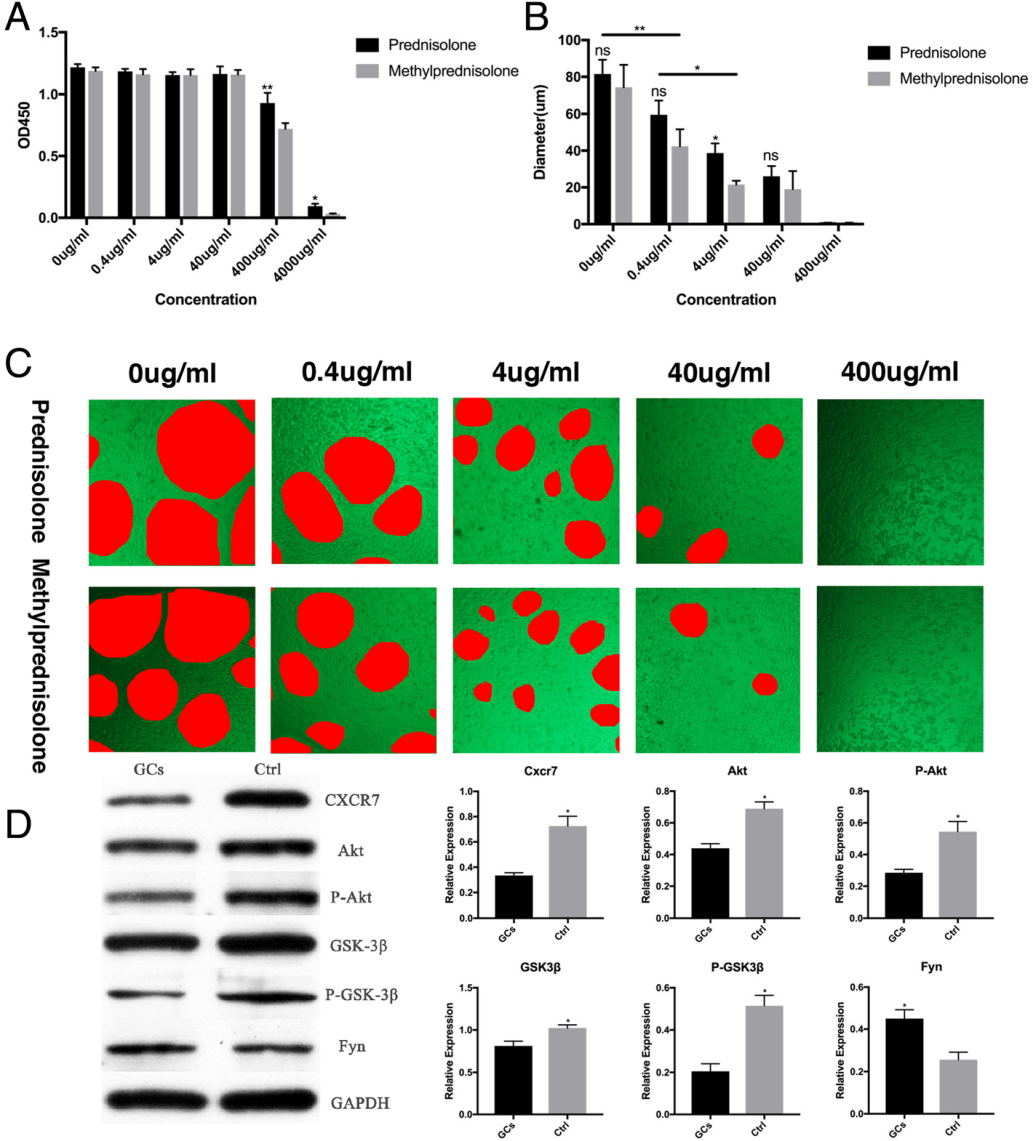
Effects of GCs on EPCs in vitro. **a** CCK8 assay showed that medium to low concentrations of GCs did not affect cells proliferation and cell growth was only suppressed under extremely high concentrations (400 μg/ml and 4 mg/ml). **b**, **c** GCs impaired the angiogenesis function of EPCs in vitro. **b** The average diameter of tubules formed by EPCs treated by different concentrations of prednisolone and methylprednisolone. **c** Representative images of tube formation assay. **d** Left panel, western blot analysis for CXCR7, Akt, phospho-Akt(P-Akt), GSK-3***β***, phospho-GSK-3***β***(P-GSK-3***β***), and Fyn in EPCs treated with GC (methylprednisolone, 40 μg/ml) or PBS (Ctrl) for 24 h. Right panel, band density analysis. *p < 0.05

Since ANFH patients had a lower number and impaired functions of circulating EPCs, we hypothesized that GCs may directly inhibit the proliferation and function of EPCs in vitro. For further investigations, cells from the healthy control group were cultured under different concentrations of two common synthetic GCs, methylprednisolone and prednisolone, independently. The number of cells was quantified by the CCK8 assay after a 72-h culture. With the concentration increasing from 0 to 40  μg/ml, the OD counts showed no significant change (Fig. 3a). The number of cells started to decrease at 400  μg/ml of GCs. At the concentration of 4  mg/ml, there was an abrupt drop of the OD reads, which suggested a significant disruption of growth of EPC at very high concentration of GCs (Fig. 3a). Our results showed that medium to low concentrations of GCs had no significant effect on cell proliferation, while cell growth was dramatically suppressed under extremely high concentrations (4 mg/ml GCs). In addition, we also found that the inhibitory effect of methylprednisolone was more significant than prednisolone at high concentrations.

Tube formation assay was also performed to evaluate the effects of GCs on EPC angiogenic function. At a lower concentration (0.4 μg/ml), both prednisolone and methylprednisolone treatments decreased the diameters of tubes formed by EPCs in vitro (Fig. 3b, c). The average diameters of tubules formed under treatments of 0.4 μg/ml GCs were close to the results obtained from ANFH patients’ EPC cultures (average diameter of tubules, 54.91 μm with prednisolone; 45.97 μm with methylprednisolone; 42.30 μm in ANFH patients as described in the “EPCs from GC-induced ANFH patients had impaired functions of migration and angiogenesis” section and Fig. 2e, f). With increased GCs’ concentration, tube formation was further suppressed. When the concentration reached 400 μg/ml, there was no neovascularization observed in each treatment group. Our results also demonstrated that at 4 μg/ml, methylprednisolone was a more potent angiogenesis inhibitor than prednisolone and the differences were statistically significant. These results suggested that the treatment of steroids significantly inhibited neovascularization by EPCs in vitro. Suppressed angiogenesis by EPCs may be an important reason for the disrupted local vascular homeostasis in GC-induced ANFH.

### Steroid treatment downregulates CXCR7 and its downstream pathway in EPCs

Our results have shown that disrupted angiogenesis was a major effect of steroids on EPCs in vitro. Emerging evidences have indicated that CXC chemokine receptor 7 (CXCR7), a receptor of SDF-1, is critical in the regulation of EPCs’ angiogenic function [16–18]. EPCs treated with oxidized low-density lipoprotein or high glucose had reduced CXCR7 expression and impaired tube formation, which was rescued by overexpression of CXCR7 [18]. The downstream Akt/GSK-3***β***/Fyn signaling pathway was critical for the pro-angiogenic function of CXCR7 in EPCs [18]. To determine whether steroids inhibit EPCs’ angiogenic function through a similar mechanism, CXCR7 expression and Akt/GSK-3***β***/Fyn pathway were analyzed. We found that after 40 μg/ml methylprednisolone treatment, CXCR7 protein level was significantly reduced (Fig. 3d). Similarly, expression of the downstream molecules Akt and GSK-3***β*** as well as their phosphorylation level were all decreased after steroids treatment (Fig. 3d). The decreases of phosphorylated Akt and GSK-3***β*** after steroids treatment were more prominent than total Akt and GSK-3***β***, suggesting that the downstream Akt/GSK-3***β*** pathway was also less activated. Increased level of Fyn was the result of downregulation of CXCR7/Akt/GSK-3***β*** pathway (Fig. 3d). These results suggested that downregulation of CXCR7 and its downstream pathway may be an important mechanism by which steroids suppress EPCs’ angiogenesis.

## Discussion

The defect of microcirculatory balance is an important mechanism for the onset and progression of GC-induced ANFH since it results in chronic regional ischemia and endothelial cell impairment in the femoral head [19–21]. Increasing evidences have shown that EPCs play a pivotal role in neovascularization and vascular repair, particularly under ischemic conditions [22, 23]. Based on these findings, emerging studies have focused on the role of dysfunction of EPCs in the pathogenesis of ANFH. Though contradictive results have been shown for the number of circulating EPCs in ANFH patients, our study further proved that in GC-induced ANFH patients, both the number and function of circulating EPCs were significantly decreased. The changes of EPCs may result in a microcirculatory imbalance and contribute to the progression of the disease. Sun et al. [5] performed EPC transplantation on rabbit glucocorticoid-induced ANFH model and found EPC transplantation combined with core decompression promoted the neovascularization and bone regeneration. Based on our and others’ studies, EPC transplantation may be a novel therapy to treat glucocorticoid-induced ANFH in the future.

Asahara et al. [3] first described human circulating angioblasts as a population of cells which were able to differentiate into epithelium cells in vitro. These so-called epithelial progenitor cells significantly contributed to neovascularization after tissue ischemia in vivo. Though markers like CD34, VEGFR2, and CD133 are typically used to identify EPC, the definition of EPC is still controversial [24]. Experimental results may be divergent when different combinations of EPC markers are used. In ANFH patients, Feng et al. [7] found that the number of CD34^+^VEGFR2^+^ cells was decreased in peripheral blood of ANFH patients. In contrast, Chen and colleagues [8] found no difference in the number of circulating CD133^+^VEGFR2^+^ cells between ANFH patients and healthy controls. Our study showed that in ANFH patients, the population of circulating CD133^+^CD34^+^cells was significantly reduced and EPCs’ functions were also impaired. By using a different combination of EPC markers, our results further proved that GC-induced ANFH patients had less circulating EPCs, which may be a major cause for the disease progression.

Migration and homing are critical for circulating EPCs to function in distal organs and ischemic tissues. Stromal cell-derived factor-1 (SDF-1) is a chemokine considered to play an important role in the recruitment of EPCs for ischemic neovascularization [25]. In our study, we for the first time used the Zigmond chamber with SDF-1 as the chemo-attractant to evaluate the migration of EPCs. Instead of measuring the number of cells moved through the pores towards the chemoattractant in transwells [7, 8], the Zigmond chamber allows accurate quantification of the cells’ migration distance and orientation by utilizing high-resolution light microscopy. By creating a SDF-1 concentration gradient, Zigmond chamber mimics the physiological environment in which circulating EPCs migrated towards distal organs in the peripheral blood. Transwell studies have shown that less EPCs migrated to VEGF in ANFH patients. By directly visualizing the trace of migration and quantification of moving distance, our study further demonstrated that EPCs’ migration ability was significantly impaired in ANFH patients when SDF1 was used as the attractant.

Though the use of steroids may affect the number and function of circulating EPCs in vivo, the direct effect of GCs on EPCs in vitro has not been extensively studied. Grisar et al. [26] showed that dexamethasone administration led to significantly increased CFU formation of EPCs in vivo. Aschbacher et al. [27] found cortisol impaired circulating angiogenic cell (CAC) migratory function and VEGF secretion in vitro. In our study, the effects of two common synthetic glucocorticoids, prednisolone and methylprednisolone, on EPCs in vitro were investigated. We demonstrated that only extremely high concentrations (0.4 and 4 mg/ml) of GCs significantly inhibited cell growth while low to medium concentrations (0.4 to 40 μg/ml) showed no effect. Since the peak serum concentrations of GCs after a high dose of oral prednisolone or pulse intravenous methylprednisolone generally range from 7 to 34 μg/ml [28], our results suggest that regular dosage of clinical steroid administration may never reach a serum concentration high enough to directly suppress the growth of EPCs. The lower number of EPCs in ANFH patients may be attributed to an indirect effect of prolonged exposure to GCs or other mechanisms. However, GCs significantly suppressed tube formation by EPCs in vitro even at low concentrations. With increased concentrations, the inhibitory effects of both synthetic GCs became more prominent. These results suggest that patients with long-term steroids treatment may have significantly suppressed neovascularization because of dysfunction of EPCs. The impaired angiogenic function of EPCs may also be an important mechanism in the progression of GC-induced ANFH.

Though GCs directly inhibit angiogenesis by EPCs in vitro, the underlying mechanism is not clear. Emerging evidences have indicated that CXCR7 is critical in the regulation of EPCs’ survival, angiogenic, and migratory functions in human and mouse [16–18, 29]. CXCR7 antagonist significantly blocked human EPCs’ tube formation in vitro [16]. In the current study, we also found that methylprednisolone downregulated the expression of CXCR7 in EPCs in vitro. Attenuated Akt/GSK-3***β*** phosphorylation and increased Fyn expression were also found after methylprednisolone treatment. Akt and GSK-3β/Fyn are important molecules downstream of CXCR7 and were responsible for the angiogenic function of EPCs [18, 30, 31]. Upregulation of Fyn may increase the degradation of Nrf2, which causes oxidative stress damage and subsequent impairment of angiogenesis [18]. These results further suggested that downregulation of CXCR7 and downstream Akt/GSK-3***β*** pathway is a novel mechanism of how GCs impair EPCs’ angiogenic function. In addition, as a receptor of SDF-1, downregulation of CXCR7 in EPCs by GCs may also explain why EPCs from GC-induced ANFH patients had decreased migratory ability when SDF-1 was used as chemo-attractant.

## Conclusion

In conclusion, our study showed that glucocorticoid-induced ANFH patients have reduced the number and impaired functions of circulating EPCs. GCs did not show a significant effect on the growth of EPCs in vitro except extremely high concentrations of GCs. However, GCs significantly impaired EPCs angiogenic function in vitro, even at low concentrations. These results indicated that EPC dysfunction may be an important reason for the development and progression of GC-induced ANFH. In addition, our study also suggested that downregulation of CXCR7 and its downstream Akt/GSK-3***β***/Fyn pathway in EPCs might be a novel mechanism of how GCs suppress EPCs’ angiogenesis.

## Data Availability

The datasets during and/or analyzed during the current study are available from the corresponding author on reasonable request.

## Abbreviations

ANFH: Avascular necrosis of the femoral head
BMI: Body mass index
BP: Blood pressure
CAC: Circulating angiogenic cell
CXCR7: CXC chemokine receptor 7
EPCs: Endothelial progenitor cells
GCs: Glucocorticoids
HC: Healthy control
MNCs: Mononuclear cells
PT: Patient
SDF-1: Stromal cell-derived factor-1
VEGF: Vascular endothelial growth factor
